# IDDoR: a novel reporter mouse system for simultaneous and quantitative *in vivo* analysis of both DNA double-strand break repair pathways

**DOI:** 10.1093/procel/pwac001

**Published:** 2022-10-14

**Authors:** Yu Chen, Zhen Cui, Zhixi Chen, Ying Jiang, Zhiyong Mao

**Affiliations:** Shanghai Key Laboratory of Maternal Fetal Medicine, Clinical and Translational Research Center of Shanghai First Maternity and Infant Hospital, Frontier Science Center for Stem Cell Research, School of Life Sciences and Technology, Tongji University, Shanghai 200092, China; Shanghai Key Laboratory of Maternal Fetal Medicine, Clinical and Translational Research Center of Shanghai First Maternity and Infant Hospital, Frontier Science Center for Stem Cell Research, School of Life Sciences and Technology, Tongji University, Shanghai 200092, China; Shanghai Key Laboratory of Maternal Fetal Medicine, Clinical and Translational Research Center of Shanghai First Maternity and Infant Hospital, Frontier Science Center for Stem Cell Research, School of Life Sciences and Technology, Tongji University, Shanghai 200092, China; Shanghai Key Laboratory of Maternal Fetal Medicine, Clinical and Translational Research Center of Shanghai First Maternity and Infant Hospital, Frontier Science Center for Stem Cell Research, School of Life Sciences and Technology, Tongji University, Shanghai 200092, China; Shanghai Key Laboratory of Maternal Fetal Medicine, Clinical and Translational Research Center of Shanghai First Maternity and Infant Hospital, Frontier Science Center for Stem Cell Research, School of Life Sciences and Technology, Tongji University, Shanghai 200092, China; Tsingtao Advanced Research Institute, Tongji University, Qingdao 266071, China; State Key Laboratory of Natural Medicines, China Pharmaceutical University, Nanjing 210009, China

**Dear Editor,**

Two distinct pathways, non-homologous end joining (NHEJ) and homologous recombination (HR) repair, have evolved to repair DNA double-strand breaks (DSBs), the most deleterious type of DNA damage. Impaired NHEJ and HR are often associated with a high incidence of tumorigenesis and the early onset of aging. A number of methods and tools have been developed to help elucidate the regulatory mechanisms of the two repair pathways in the past. *In vitro* biochemical assays employ ^32^P-labeled broken DNA fragments and cell extracts to evaluate DSB repair capacity. At DSB sites, several DSB repair factors form foci, which can be visualized through immunofluorescence and microscopy. Among them, phosphorylated H2AX at the Ser139 residue is often considered a DSB marker, and its formation and removal at different time points post the induction of DSBs can be used to reflect the repair capacity and genomic instability. In addition, by quantifying the kinetics of the recruitment of specific NHEJ and HR factors such as 53BP1, DNA-PKcs, MRE11, RPA2, and RAD51, one would be able to make judgments on whether NHEJ, HR, or both pathways is/are affected and sometimes at which steps DSB repair pathways are regulated. An alternative approach for kinetic analysis is to quantify the microirradiation-induced recruitment of repair factors. Moreover, comet assays are also utilized to measure DNA damage-induced genomic stability at the single-cell level. However, with these assays, it is still difficult to measure NHEJ and HR efficiency in a relatively less time-consuming and more quantitative manner.

With the discovery of fluorescent proteins and the development of flow cytometry technology, several groups have created a number of fluorescence protein-based reporters to rapidly and quantitatively measure NHEJ and HR efficiency (Pierce et al., 1999; Seluanov et al., 2004; Bennardo et al., 2008; Mao et al., 2008, 2012; Certo et al., 2011; Bindra et al., 2013; Gomez-Cabello et al., 2013; Chen et al., 2019). For instance, for measuring NHEJ efficiency, the pEGFP-pem1-Ad2 vector and EJ5-GFP vector are widely used (Seluanov et al., 2004; Bennardo et al., 2008), and DR-GFP reporter is the most well-established reporter for the analysis of HR (Pierce et al., 1999). Other reporter cassettes to measure short-tract HR, long-tract HR, gene conversion, crossover, and single-strand annealing pathways have also been developed (Mao et al., 2008, 2012). Recently, several groups created different reporter cassettes for simultaneously measuring both NHEJ and HR at the same chromosomal sites (Certo et al., 2011; Gomez-Cabello et al., 2013; Chen et al., 2019). Together with other approaches for studying DSB repair, these reporters have greatly advanced our knowledge on the regulatory mechanisms of DSB repair by NHEJ and HR using cellular models.

Recently, three different knock-in mouse models were generated to measure NHEJ or HR efficiency *in vivo*. Among them, one has an NHEJ reporter knocked into the *Rosa26* locus (*Rosa26*^NHEJ/+^) and the other two have HR reporters integrated into the *Pim1* locus (*Pim1*^DR-GFP/+^) or *Rosa26* locus (*Rosa26*^HR/+^) (Kass et al., 2013, 2016; Vaidya et al., 2014; Wang et al., 2020). The endonuclease I-SceI recognition sites were introduced to the three vectors to enable the induction of DSBs upon the expression of I-SceI. By hydrodynamic tail vein injection, sufficient amounts of I-SceI expression vectors were introduced to livers to induce DSBs on the reporters, thereby allowing the analysis of the efficiency of DSB repair by NHEJ or HR in livers (Wang et al., 2020). A transgenic mouse model with the pTRE-I-SceI vector was also created to breed with pCMV-rtTA transgenic mice and *Pim1*^DR-GFP/+^ mice. In bred mice fed doxycycline-supplemented water, successful HR events were detected in mammary, bone marrow, and intestinal tissues (Kass et al., 2016). However, various aspects need to be improved to optimize the *in vivo* analysis of DSB repair. The currently available mouse models do not allow the analysis of NHEJ or HR efficiency simultaneously, and we cannot compare the efficiency of the two pathways at the same chromosomal sites. In addition, other than the four organs in which DSB repair efficiency can be calculated, whether NHEJ and HR events can also be quantitatively scored in more organs or tissues remains to be determined. Moreover, whether these mouse models can be further modified to simplify the breeding process needs to be investigated.

In the present study, we successfully designed, generated, and validated a novel reporter mouse system (IDDoR: inducible dual-fluorescence-based double-strand break repair reporter). The IDDoR system comprises two mouse models that can be combined to simultaneously analyze both NHEJ and HR efficiency at the same chromosomal sites in a variety of types of tissues.

To generate the IDDoR system, we first created a reporter mouse, namely, Fireworker, containing the dual-fluorescence reporter cassette at the *Rosa26* site. The targeting vector comprises two major parts. The first part contains one rat Pem1 intron interrupted by four engineered exons: mouse HPRT exon 1 with an engineered splicing acceptor (SA) site and the start codon ATG before the splicing donor (SD) site, two I-SceI recognition sites flanking adenoviral exon 2 with one more nucleotide inserted (Ad+1nt), an ATG-less tdTomato gene with no SD or SA sites, and an ATG-less GFP gene with SA site inserted before itself (Fig. 1A). The second part is composed of a partial Pem1 intron and a partial tdTomato gene with an engineered SA site (Fig. 1A). The targeting vector was introduced into embryonic stem (ES) cells derived from C57BL/6 × 129/Sv mice. The successfully targeted vector was integrated into the site downstream of the *Rosa26* promoter and *Rosa26* exon 1 (Fig. 1A). The targeted genome was validated through Sanger sequencing of the amplified homologous arms and through long-range PCR of the vector (Fig. S1A–D).

**Figure 1.**
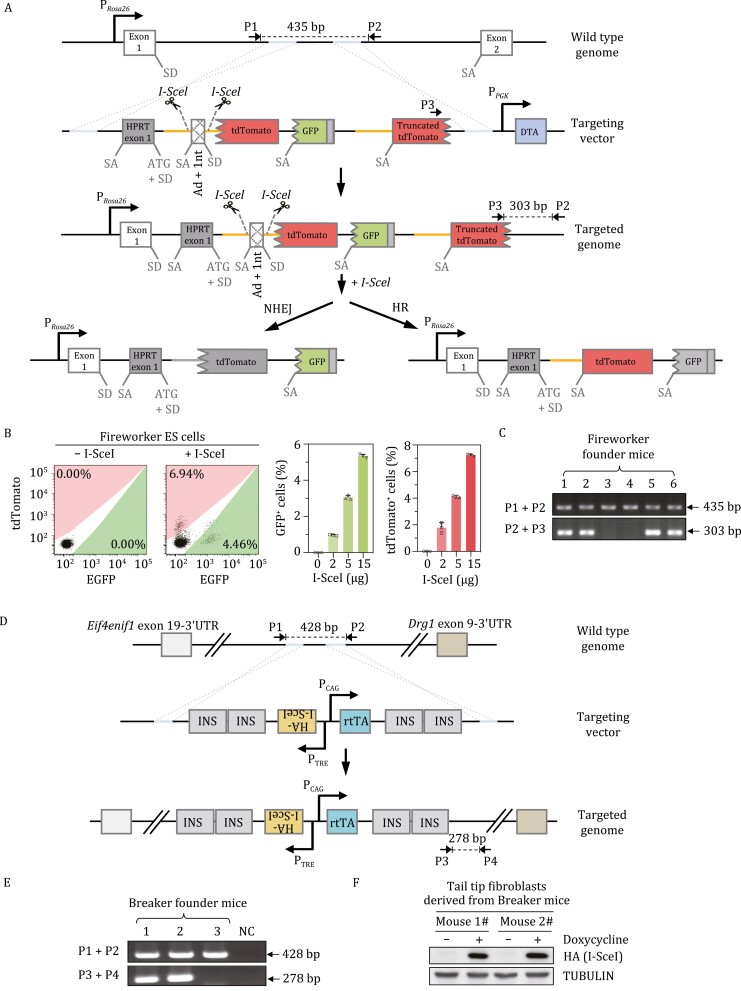
Establishment of knock-in reporter mice for simultaneous *in vivo* analysis of NHEJ and HR efficiency. (A) Schematic diagram of the targeting strategy at the *Rosa26* locus for establishment of the Fireworker mouse model. The dual-fluorescence-based reporter cassette functions to simultaneously measure NHEJ and HR efficiency *in vivo* were designed based on the previously published dual-fluorescence reporter for *in vitro* efficiency analysis. The orange line represents the 5ʹ homologous sequence, and the tdTomato ORF is the 3ʹ homologous sequence. The broken line at the 5ʹ end of tdTomato and GFP ORF stands for the lack of start codon. Ad, adenoviral exon; SD, splice donor; SA, splice acceptor. NHEJ contributes to GFP expression, while HR repair leads to tdTomato expression. (B) FACS analysis of GFP^+^ and tdTomato^+^ cells in successfully targeted mouse ES cells at 48 h post transfection with different amounts of pCAG-HA-I-SceI vector. Representative FACS traces are shown in the left panel, and the percentages of GFP^+^ and tdTomato^+^ cells are shown in the right panel. Error bars represent the SD. (C) Genotyping with genomic DNA extracted from the tail tips of newborn founder mice. The primers used were as indicated in (A). (D) Schematic diagram of the targeting strategy at the *H11* locus for establishment of the Breaker mouse model. The targeting vector was modified from our previously published doxycycline-inducible I-SceI expression vector. (E) Genotyping with genomic DNA extracted from the tail tips of newborn founder mice. The primers used were as indicated in (D). (F) The expression of HA-tagged I-SceI was analyzed by Western blot in primary tail tip fibroblasts derived from two different knock-in mice with or without doxycycline supplementation.

In the absence of I-SceI expression, the three engineered fluorescent genes were all negative. In theory, the first ATG-less tdTomato gene cannot be spliced into the mature mRNA due to the absence of SD and SA. The GFP gene can be spliced into the mature mRNA, but the 1 nt insertion in the Ad exon completely disrupts the GFP gene. Since there was no additional SD after the GFP gene and the second tdTomato gene lacked 677 bp at the 3ʹ end, the second tdTomato could not be correctly expressed and was also fluorescence negative. Instead, upon the removal of Ad + 1 nt exon and the induction of DSBs by endonuclease I-SceI digestion, successful NHEJ led to the expression of the functional GFP gene after transcription and splicing. In contrast, successful HR would result in the HR product, which could be turned into a functional full-length tdTomato (Fig. 1A).

To assess the above assumption, we electroporated the ES cells harboring Fireworker reporter with pControl and pCAG-I-SceI vectors and then performed FACS analysis. We observed no GFP^+^ or tdTomato^+^ cells in control vector-transfected ES cells, while transfecting increasing amounts of pCAG-I-SceI vectors led to a gradual percentage increase in both GFP^+^ or tdTomato^+^ ES cells (Fig. 1B). Moreover, the tdTomato^+^ cells were sorted for genomic DNA extraction, and the repaired DSB region was amplified for Sanger sequencing (Fig. S1E). The sequencing results, which revealed the acquirement of ATG start codon before the ATG-less tdTomato gene, confirmed that the expression of tdTomato was the consequence of successful HR (Fig. S1F). To validate Fireworker reporter can correctly reflect the efficiency of NHEJ and HR, we knocked down critical genes regulating NHEJ or HR in ES cells or treated ES cells with small molecules targeting DSB repair factors and then examined the efficiency of NHEJ and HR by transfecting cells with vectors expressing I-SceI and an internal control pCAG-mTagBFP2 to normalize transfection efficiency. NHEJ can be further categorized into two subpathways: canonical NHEJ (c-NHEJ) and alternative NHEJ (alt-NHEJ), and the two subpathways compensate for each other. We found that indeed depleting either KU80, the essential c-NHEJ factor, or PARP1, the essential alt-NHEJ factor, had no significant effect on NHEJ efficiency measured by the ratio of GFP^+^/mTagBFP2^+^, while knocking down both factors significantly reduced NHEJ efficiency by 44.3% (Fig. S2A). We also found that knocking down RAD51, the major HR recombinase, or CtIP, the critical end resection factor, led to a 64.8% or 54.7% reduction in HR efficiency measured by the ratio of tdTomato^+^/mTagBFP2^+^ (Fig. S2B). Moreover, blocking the enzymatic activity of the c-NHEJ factor DNA-PKcs with Nu7026 or suppressing the catalytic activity of the alt-NHEJ factor PARP1 with olaparib had no significant effect on NHEJ, but the simultaneous inhibition of the catalytic activity of both enzymes strongly inhibited the NHEJ efficiency by 75.3% in ES cells (Fig. S2C). In addition, treating cells with mirin, a small molecule blocking MRE11 endonuclease activity, drastically reduced HR efficiency by 85.8% in the ES cells (Fig. S2D). Taken together, these data indicate that we successfully obtained ES cells with the chromosomally integrated Fireworker reporter that can simultaneously measure both NHEJ and HR at the same chromosomal sites.

Next, *Rosa26*^Fireworker/+^ ES cells were injected into blastocysts to generate chimeric mice, which were further mated with wild-type C57BL/6 mice. The founder mice were genotyped with the two sets of indicated primers. The genotyping results indicated that we successfully obtained the knock-in mouse model with the Fireworker reporter integrated at the *Rosa26* site (Fig. 1C). In addition, to rule out the possibility that multiple copies of the reporter cassettes were integrated to chromosomal positions other than the *Rosa26* site, we performed quantitative PCR to assess the copy number of the reporter in the founder mouse with the previously reported *Rosa26*^NHEJ/+^ and *Rosa26*^NHEJ/NHEJ^ reporter mouse model as the reference (Vaidya et al., 2014). We found that the *Rosa26*^Fireworker/+^ founder mouse had only one copy of reporter cassette integrated into the genomes (Fig. S1G).

To evaluate the *in vivo* DSB repair capacity using the *Rosa26*^Fireworker/+^ mouse, the mouse model with I-SceI expression under an inducible promoter needs to be created and bred with the *Rosa26*^Fireworker/+^ mouse. In a previous report using the DR-GFP reporter mouse model, the doxycycline inducible I-SceI system with two transgenic mouse models, the mouse model harboring the pTRE-I-SceI vector and the one containing the pCMV-rtTA vector, was employed. To simplify the breeding process, we created the *H11* site targeting vector combining the two-expression apparatus: pTRE-HA-I-SceI and pCAG-rtTA. To avoid potential interference with each other, the two units containing the promoter and the downstream gene were placed in an opposite orientation (Fig. 1D). We also inserted two tandem insulators into the 5ʹ and 3ʹ ends to prevent the potential influence of flanking *cis*-regulatory elements on rtTA or I-SceI transcription (Fig. 1D). The vector was injected into C57BL/6J zygotes along with the CRISPR–Cas9 vectors. The obtained founder mice, namely, Breaker, were genotyped with the indicated primers (Fig. 1E) and validated by Sanger sequencing of both homologous arms and by long-range PCR (Fig. S3A–C). In addition, quantitative PCR was also performed to validate that only one copy of the targeting vector was inserted into the genome by referring to the genomes of well-characterized *Rosa26*^rtTA/+^ and *Rosa26*^rtTA/rtTA^ mice (Hochedlinger et al., 2005) (Fig. S3D). Moreover, we also isolated fibroblasts from the tail tips of founder Breaker mice. We treated fibroblasts with doxycycline, followed by Western blot analysis of I-SceI expression. We observed strong expression of I-SceI in response to doxycycline supplementation (Fig. 1F).

To test the feasibility of analyzing NHEJ and HR *in vivo*, we bred the *Rosa26*^Fireworker/Fireworker^ mouse with the *H11*^Breaker/Breaker^ mouse and obtained the *Rosa26*^Fireworker/+^*H11*^Breaker/+^ mouse (IDDoR mouse). The IDDoR mice were fed doxycycline-supplemented drinking water for nine consecutive days, followed by Western blot analysis of I-SceI expression in different organs (Fig. 2A). We observed the expression of I-SceI in a variety of organs and tissues, including the kidney, brain, heart, intestine, pancreas, stomach, and skin (Fig. 2B). Taken together, these results suggest that the *H11*^Breaker/+^ mouse model was successfully generated for controllable *in vivo* I-SceI expression. Moreover, I-SceI expression was also validated by immunofluorescence experiments. By staining the samples with an antibody against HA, we confirmed the expression of I-SceI in the seven tissues (Fig. 2C). Next, by costaining the samples with antibodies against GFP and tdTomato at 21 days post doxycycline administration, we observed GFP^+^ cells in all seven organs with I-SceI expression, suggesting that NHEJ operates in all seven organs (Fig. 2D). In contrast, we only observed tdTomato^+^ cells in four organs: pancreas, stomach, intestine, and skin, indicating that HR-directed repair occurs in the four organs (Fig. 2D). To further validate that the expression of I-SceI is tightly controlled by the TRE promoter and whether the potential leakage of I-SceI would lead to the accumulation of fluorescent cells, 1-year-old IDDoR mice were sacrificed for GFP and tdTomato staining in the seven organs. The results showed that there was no detectable GFP and tdTomato signal in these organs (Fig. S4), suggesting that IDDoR mice can also be employed for long-term studies, such as age-related regulation of DNA repair. Altogether, these data demonstrate that the IDDoR mouse system can be used to quantify GFP^+^-reflected NHEJ events and tdTomato^+^-reflected HR events. Moreover, the IDDoR mouse system would provide a versatile tool to elucidate the underlying mechanisms that regulate the organ-specific, and cell type-specific usage of HR, NHEJ and the choice of the two pathways in different biological contexts such as aging, cancer, and other types of diseases.

**Figure 2.**
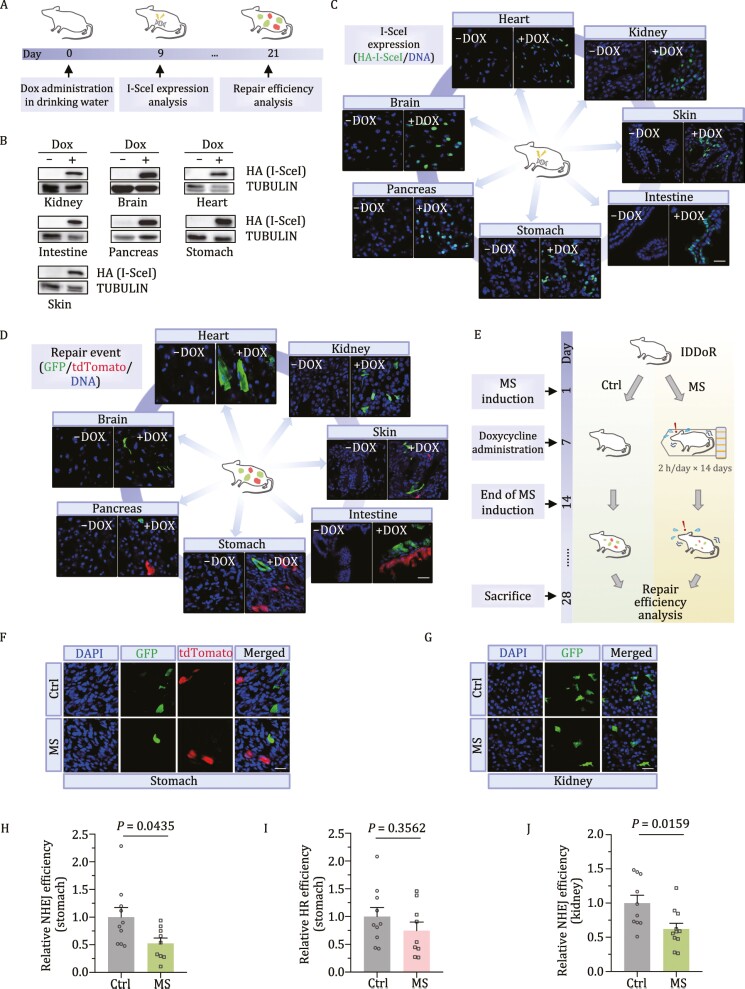
*In vivo* analysis of DSBR efficiency employing the IDDoR system. (A) Diagram of controllable *in vivo* DSB induction and repair efficiency analysis. IDDoR mice were administered 3 mg/mL doxycycline in drinking water with 5% sucrose. The mice were sacrificed at day 9 and day 21 to dissect different organs for analyzing I-SceI expression and repair events, respectively. (B and C) The expression of HA-tagged I-SceI was analyzed by Western blot and immunofluorescence assay in different tissues at day 9. (D) GFP^+^ and tdTomato^+^ cells, which represent successful NHEJ and HR events, respectively, were analyzed by immunofluorescence assay at day 21. (E) Experimental procedure for analyzing the influence of mental stress on NHEJ and HR in the stomach and kidney employing the IDDoR system. The IDDoR mice were randomly grouped. Mice in the MS group were restrained in 50 ml plastic centrifuge tubes individually, while the control mice were left in the cages and allowed for free activities and contact with each other but also with no access to food and water for 2 h/day from day 1 to day 14. Doxycycline was added to the drinking water from day 7. Mice were sacrificed at day 28 for repair efficiency analysis. (F and G) Representative images of GFP^+^ and tdTomato^+^ cells in the stomach and kidney. (H and I) NHEJ and HR efficiency in the stomach of control mice and mice with MS. NHEJ efficiency was measured by the ratio of GFP^+^ cells/I-SceI expression level, and HR efficiency was measured by the ratio of tdTomato^+^ cells/I-SceI expression level. *n* = 10 mice for the control group, and *n* = 9 mice for the MS group. (J) NHEJ efficiency in the kidneys of control mice and mice under MS. NHEJ efficiency was measured by the ratio of GFP^+^ cells/I-SceI expression level. *n* = 10 mice for the control group and *n* = 11 mice for the MS group. Ctrl, control mice; MS, mice with mental stress. Error bars represent the SEM. The Mann-Whitney U test was employed for significance determination. Scale bar: 20 μm. The quantification data of GFP^+^ and tdTomato^+^ cells and I-SceI expression level are included in Fig. S5S.

Psychological stress has been linked with the onset of aging and aging-associated diseases. Although genomic instability, which can arise from impairment in DNA repair, is a fundamental hallmark of aging and aging-associated diseases, such as cancer, the clear relationship between mental stress and DNA repair has not yet been well defined due to the lack of appropriate research tools. Several studies have indicated that the level of DNA damage, including strand breaks, is elevated in the brain, the main organ involved in mental stress sensing, implying a decline in DNA repair capacity in the brain. Nevertheless, whether mental stress also influences DNA repair in other organs remains unclear. Therefore, one of the major advantages of using the IDDoR mouse model over the cellular models and *ex vivo* DNA repair assays is that it can be used to study how mental stress affects DSB repair efficiency in different organs other than brains. For instance, psychological stress causes changes in stomach function and promotes gastric disease development, and it is also associated with kidney diseases. We, therefore, employed IDDoR mice to examine whether mental stress affects DSB repair efficiency in the stomach and kidney (Fig. 2E). For 2 h/day, the IDDoR mice were individually placed inside 50-ml plastic centrifuge tubes with small punctures for 14 consecutive days to develop mental stress as previously reported (Tye et al., 2013). From the second week of stress induction, these mice and control mice were fed doxycycline-supplemented drinking water for 21 days before being sacrificed for stomach and kidney harvesting. The harvested organs were further processed into frozen sections for coimmunostaining with antibodies against tdTomato and GFP (Fig. 2E). We successfully observed GFP^+^ and tdTomato^+^ cells in the stomach sections and only GFP^+^ cells in the kidney sections (Figs. 2F, 2G, S5A, S5B, and S5H). Since I-SceI expression might be unidentical between mice, it is essential to calculate the relative I-SceI expression level for the normalization of HR and NHEJ efficiency (Fig. S5C, S5D, S5I, and S5J). Then, we used the ratio of the percentage of GFP^+^ cells/I-SceI expression level as the measurement of NHEJ efficiency, and the ratio of the percentage of tdTomato^+^ cells/I-SceI expression level as the measurement of HR efficiency.

By comparing DSB repair efficiency in the stomachs between the control and mental stress groups, we surprisingly found that mental stress significantly suppressed NHEJ efficiency by 47.4%, while HR efficiency also showed a trend of decline, although not significant, in stomachs (Figs. 2H, 2I and S5L). Moreover, NHEJ efficiency was also found to decrease by 38.0% in the kidneys of mice with mental stress compared to the control group (Fig. 2J). HR only occurs in the S/G_2_ phase when sister chromatids are available, we therefore also stained frozen stomach sections with Ki67, a widely used proliferation marker, and quantified the percentage of Ki67^+^ cells to rule out the possibility that the analysis of HR efficiency was influenced by the alteration in cell proliferation rates (Fig. S5E and S5F). In addition, quantitative PCR was performed to validate that the decline in DSB repair capacity was not attributed to differences between the transcription levels of the genes under the *Rosa26* promoter (Fig. S5G and Fig. S5K). These results demonstrated that mental stress has an interorgan influence on the DSB repair capacity in other organs, such as stomachs and kidneys.

The analysis of DSB repair efficiency using a variety of types of fluorescence-based reporter cell lines has been widely used to elucidate the regulatory mechanisms of DSB repair by either NHEJ or HR (Pierce et al., 1999; Seluanov et al., 2004; Bennardo et al., 2008; Mao et al., 2008, 2012; Certo et al., 2011; Bindra et al., 2013; Gomez-Cabello et al., 2013; Chen et al., 2019). In addition, cellular models harboring reporter cassettes can also be applied to screen small molecules targeting DSB repair, thereby laying the foundation for developing novel methods of treating cancer, delaying the onset of aging, and improving genome editing efficiency. However, since most of organs are composed of a variety of different types of cells, whether the analysis of DSB repair efficiency in the *in vitro* cultured single types of cells can reflect the real *in vivo* situation remains to be determined. In addition, numerous scientific questions cannot be correctly and clearly answered with the currently widely used DNA repair assays in cultured mammalian cells. For examples, is there an inter-organ communication in DSB repair? Can emotion, caloric restriction, physical activity, circadian rhythm, and etc. affect DSB repair, and do they have tissue-specific effects? Moreover, not all types of cells can be long-term cultured *in vitro*, so assessing the repair efficiency in these types of cells in different biological contexts is extremely technically challenging. For instances, the difficulty in culturing cardiomyocytes is one of the major reasons that hampers the analysis of the correlation between DSB repair efficiency and heart diseases such as cardiac hypertrophy or cardiac fibrosis.

Fortunately, several reporter mouse models have been developed and employed to study DSB repair in different biological contexts. HR events reflected by GFP^+^ cells were observed in three different proliferating tissues, mammary gland, bone marrow, and intestine, in the I-SceI DR-GFP mice derived from three mouse lines: DR-GFP mouse, pTRE-I-SceI mouse and pCMV-rtTA mouse. Using a mouse model, the indispensable role of BRCA2 in HR was confirmed, suggesting that *in vivo* models can be utilized to validate cell culture-based discoveries (Kass et al., 2016). However, the I-SceI DR-GFP mice require a time-consuming mating process, and it can only score HR in a certain number of tissues. Another successful application using DSB repair mouse models is the analysis of NHEJ and HR efficiency in the livers of *Rosa26*^NHEJ^ and *Rosa26*^HR^ mice (Wang et al., 2020). By introducing large amounts of pCMV-I-SceI vectors into the liver through hydrodynamic tail vein injections, the DSB repair efficiency was compared between normal liver tissues and hepatocellular carcinoma tissues using the two DSB repair mouse models. The observed difference in DSB repair efficiency between normal and tumor tissues enables further mechanistic and preclinical studies. However, this tool can only be used to assess HR or NHEJ efficiency in livers. Our newly developed IDDoR mouse system expands the list of organs in which both DSB repair efficiencies can be quantitatively scored. The IDDoR mouse system simplifies the mating process, as only *Rosa26*^Fireworker/Fireworker^ mice and *H11*^Breaker/Breaker^ mice need to be bred. Another advantage of the IDDoR mouse system is that it can simultaneously analyze both NHEJ and HR efficiency, and the choice between NHEJ and HR can also be assessed at the defined chromosomal sites. The successful analysis of NHEJ and HR in two organs in control mice and mice with mental stress in this study indicates that the IDDoR system can be further applied to study DSB repair in a variety of biological contexts.

Nevertheless, it is still laborious and time-consuming to obtain desired mice for analysis when one needs to study how knocking out a gene affects *in vivo* DSB repair in certain tissues. Whether it is feasible to combine the Fireworker reporter and the Breaker vector on the same chromosome to further simplify the IDDoR system warrants further investigation. For instance, could the doxycycline inducible I-SceI vector inserted after the dual-fluorescence reporter cassette be still functional? In addition, feeding IDDoR mice doxycycline-supplemented drinking water simultaneously induced the expression of I-SceI in a number of tissues, causing DSBs at the *Rosa26* sites in the cells of those tissues. Although mice might be capable of tolerating one DSB induced by I-SceI in each cell, it would be intriguing to create a tissue-specific and temporally I-SceI-inducible mouse model. For instance, it would be interesting to convert the inducible CreER (Tam) system into the inducible I-SceIER system. Moreover, there still exist several issues which can be optimized to improve our reporter. For instance, I-SceI has been reported to be bound to cleave DNA, and it may potentially impact the DNA repair process. Although, the reporter has been validated with RNAi and inhibitors of well-known repair factors (Fig. S2), creating a novel engineered I-SceI mutant that retains its endonuclease activity but abolishes its high affinity to DNA ends would make the I-SceI-generated DNA damage more representative of the endogenously or exogenously generated DSBs. Another issue is that unrepaired DSBs may cause cell death, and it may result in the underestimation of the total cell number, and create a bias for repair efficiency calculation. Therefore, cell survival may be another parameter which should be included in the analysis of repair efficiency.

In summary, we designed, generated and characterized a novel reporter mouse system—IDDoR—that can be utilized for the simultaneous analysis of both NHEJ and HR efficiency at defined genomic loci in different types of organs. The system can be further applied in studying the connections of DSB repair to numerous biological processes, such as tumorigenesis, the onset of aging, and social behaviors *in vivo*.

## Supplementary Material

pwac001_suppl_Supplementary_Material

## Footnotes

The work was supported by grants from the Chinese National Program on the Key Basic Research Project (2018YFC2000100 and 2021YFA1102000 to Z.M.), the National Natural Science Foundation of China (31871438 and 82071565 to Z.M., 81972457 and 32171288 to Y.J.), the “Shu Guang” project supported by the Shanghai Municipal Education Commission and Shanghai Education Development Foundation (19SG18 to Z.M.), the Program of the Shanghai Academic Research Leader (19XD1403000 to Z.M.), and the Shanghai Sailing Program (22YF1434300 to Y.C.).

Y.C., Z.C., Z.C., Y.J. and Z.M. declare no competing interests..

All data generated or analysed during this study are included in this published article (and its supplementary information files).

Y.C., Z.C. and Z.C. performed experiments and analyzed data. Y.C., Y.J. and Z.M. were involved in the conception and design and data interpretation. Y.C. and Z.M. wrote the manuscript. Z.M. was responsible for the final approval of manuscript.
