# Supplementary material for: IDDoR: a novel reporter mouse system for simultaneous and quantitative in vivo analysis of both DNA double-strand break repair pathways
Source: Protein Cell. 2022 Oct 14;14(5):369–75. doi: 10.1093/procel/pwac001 (PMC10166171; doi:10.1093/procel/pwac001)
Supplement: pwac001_suppl_Supplementary_Material [file pwac001_suppl_supplementary_material.pdf]

Supplementary Materials

IDDoR: A novel reporter mouse system for simultaneous and quantitative *in vivo* analysis of both DNA double-strand break repair pathways

Yu Chen[1], Zhen Cui[1], Zhixi Chen[1], Ying Jiang[1, *], Zhiyong Mao[1, 2, 3, *]

[1] Shanghai Key Laboratory of Maternal Fetal Medicine, Clinical and Translational Research Center of Shanghai First Maternity and Infant Hospital, Frontier Science Center for Stem Cell Research, School of Life Sciences and Technology, Tongji University, Shanghai 200092, China
[2] Tsingtao Advanced Research Institute, Tongji University, Qingdao 266071, China
[3] State Key Laboratory of Natural Medicines, China Pharmaceutical University, Nanjing 210009, China

[*] To whom correspondence should be addressed: Ying Jiang & Zhiyong Mao. Tel: +86-21-65978166; Fax: +86-21-65981041; Email: ying_jiang@tongji.edu.cn; zhiyong_mao@tongji.edu.cn.

Materials and Methods

Animals

C57BL/6 mice were housed in a specific pathogen-free (SPF)-grade environment. All mice were maintained under a 12-hour light/dark cycle and had free access to food and water. All experiments were performed in accordance with the Health Guide for the Care and Use of Laboratory Animals and were approved by the Biological Research Ethics Committee of Tongji University.

Generation of dual fluorescence-based reporter mice (*Fireworker* mice)

The targeting vector was modified from a previously reported NHEJ-I vector. Briefly, an ATG-less tdTomato ORF was inserted to the site downstream of the second exon of GFP. Then, the first exon of GFP was replaced with the HPRT exon, and the second exon of GFP was replaced with the ATG-less GFP ORF. A single nucleotide was then added to Ad, SA was inserted into the 5' end of the HPRT exon, and the starting codon, ATG, was inserted into the 3' end of the HPRT exon. Finally, the modified repair cassette was digested and ligated to the backbone of a previously published *R26*NHEJ targeting vector (Vaidya et al., 2014). The primers used for genotyping were as follows: P1-5' GGGGCGTGCTGAGCCAGACCTCCAT 3'; P2-5' TCCCGACAAAACCGAAAATCTGTGG 3'; P3-5' TGCATCGCATTGTCTGAGTAGG 3'. The wild-type allele can be amplified by P1 and P2, generating a 435 bp band, and the knocked-in allele can be amplified by P2 and P3, generating a 303 bp band.

Generation of inducible I-SceI mice (*Breaker* mice)
The targeting vector was modified from a previously reported inducible I-SceI expression vector. Two tandem insulators were added upstream and downstream of the inducible I-SceI expression element by in-fusion cloning to avoid any potential influence of flanking sequences. The targeting vectors contained a 5 kb 5' homologous arm and a 3.8 kb 3' homologous arm targeting the mouse *H11* locus. The vector was microinjected along with the *in vitro* transcribed Cas9 mRNA and gRNA into C57BL/6 zygotes to generate founder mice. The primers used for genotyping were as follows: P1-5' ATAAGCCATTCTCCATTTCATAA 3'; P2-5' CCCCTTGTTCCCTTTCTGC 3'; P3-5' CCGTCGAGGCTTGGGTGATA 3'; P4-5' CATGATTAGTGTTTGCCTTTGTTA 3'. The wild-type allele can be amplified by P1 and P2, generating a 428 bp band, and the knocked-in allele can be amplified by P3 and P4, generating a 278 bp band.

***In vivo* analysis of DNA double-strand break repair efficiency**
Rosa26$^{\mathrm{Fireworker/+}}$ mice were mated with *H11*$^{\mathrm{Breaker/+}}$ mice to obtain IDDoR mice. The IDDoR mice were fed water supplemented with 3 mg/mL doxycycline and 5% sucrose for 3 weeks and then sacrificed to dissect different organs, including the heart, kidney, pancreas, stomach, intestine, brain and skin. Organs were immediately fixed with 4% paraformaldehyde for immunofluorescence or frozen at -80°C for genomic DNA and RNA extraction. For each mouse, at least six frozen sections were counted for analyzing GFP, tdTomato and Ki67 positive cells.

Mental stress induction
Mental stress was induced using a restraint model as previously reported (Tye et al., 2013; Wu et al., 2016; Zhang et al., 2020). Briefly, 6-week-old mice were individually placed inside 50 mL plastic centrifuge tubes with small punctures for two hours/day. Six-week-old control mice were maintained away from food and water but allowed free activities and contact with each other in their original cages for two hours/day. The induction period lasted for a total of 14 consecutive days.

Immunofluorescence histochemistry
The freshly dissected tissues were fixed with 4% paraformaldehyde at 4 °C overnight, washed with PBS 3 times and treated with 30% sucrose/PBS solution for cryopreservation until the tissues sank. Then, the tissues were embedded in Tissue-Tek® O.C.T. compound and sectioned with Leica CM1950 cryostats. The sections were washed with PBS three times and incubated in 0.5% Triton-X-100/PBS solution for 10 minutes. After rinsing with PBS 3 times, the sections were blocked with 2% BSA/PBS for 1 hour at room temperature and then incubated with primary antibodies at 4 °C overnight. The next day, the sections were rinsed with PBS 3 times and incubated with secondary antibodies at room temperature for 1 hour. The sections were then covered with mounting medium containing DAPI nuclear staining followed by PBS washing 3 times. For each mouse, at least six frozen sections were counted.

Cell culture and transfection

The mouse ES cells were maintained on mitomycin C-treated mouse embryonic fibroblasts in DMEM supplemented with 15% fetal bovine serum, 1 × nonessential amino acids, 1,000 U/mL LIF, 1 mM L-glutamine, 1 × nucleoside, 0.1 mM 2-mercaptoethanol, 1 μM PD0325901, 3 μM CHIR-99021 and 1% penicillin/streptomycin. Primary mouse tail-tip fibroblasts were maintained in DMEM supplemented with 10% fetal bovine serum, 1 × nonessential amino acids and 1% penicillin/streptomycin. All cells were cultured in a 5% CO_2 and 3% O_2 humidified incubator (Thermo Fisher HERAcell 240i) at 37 °C. Mouse ES cells were electroporated on a Lonza 4D machine with CG-104 program.

Antibodies and siRNAs

The antibodies used in this study were as follows: anti-HA (Cell Signaling, Cat. #3724), Anti-GFP (Aves lab, Cat. #GFP-1020), Anti-tdTomato (Rockland, Cat. #600-401-379), Anti-Ki67 (Thermofisher, Cat. #MA5-14520), Anti-GAPDH (Proteintech, Cat. #60004), Anti-Rabbit IgG-HRP (Bio–Rad, Cat. #170-6515), anti-chicken IgY-Alexa Fluor® 488 (Jackson ImmunoReaearch, Cat. #703-545-155), anti-rabbit IgG-DyLight 594 (Invitrogen, Cat. #SA5-10040), anti-γH2AX (S139) (Cell Signaling, Cat. #9718), Anti-β-TUBULIN (Bioworld, Cat. #AP0064).

The siRNA sequences against *Rad51*, *CtIP*, *Ku80* and *Parp1* were as follows: si*Rad51*-5' CGGUCAGAGAUCAUACAGAUATT 3'; si*CtIP*-5' GGAACUCUGGACAAAACUATT 3'; si*Ku80*-5' UACGACAACUGUGCGUCUUUATT 3'; and si*Parp1*-5' CCAAAGGAAUUCCGAGAAATT 3'.

Real-time PCR

For copy number determination of the *Fireworker* vector, genomic DNA was prepared from the mouse tail tip and amplified with primers against the GFP ORF. The sequences were as follows: F-5' TTTCCAAGAAGCTTTAGGGA 3'; R-5' CGAACCAGTTCTACATGCTA 3'. For copy number determination of the *Breaker* vector, tail tip genomic DNA was amplified with primers against the rtTA ORF. The sequences were as follows: F-5' TCGCGACGGGGCTAAAGTGC 3'; R-5' TGGGGGCATAGAATCGGTGG 3'. To analyze the transcriptional level of the *Rosa26* locus, total RNA was extracted from stomach and kidney tissue with an RNAsimple kit (TIAGEN, Cat. #DP419) and reverse transcribed with TransScript® One-Step gDNA Removal and cDNA Synthesis SuperMix (Trans, Cat. #AT311-02). cDNA was used as a template for real-time PCR with the following primers: F-5' GCCTAAAGAAGAGGCTGTGC 3'; R-5' CGGCTGTCTCACAGAACG 3'. GAPDH was amplified with F-5' ATGACATCAAGAAGGTGGTG 3'; R-5' CATACCAGGAAATGAGCTTG 3' and used as an internal control.

Statistical analysis

GraphPad Prism 8 software was applied for statistical analysis. Sample size was determined on the basis of experience with similar experiments and from that was generally used in other studies. Data were expressed as the mean ± s.d. unless indicated otherwise. Unpaired Student's t-test was employed to determine whether there was a significant difference between two groups. For animal studies, statistical evaluations of *in vivo* repair

efficiency, I-SceI expression level, percentage of Ki67^{+} cells and transcription level were carried out with the Mann–Whitney U test.

References

Tye, K.M., Mirzabekov, J.J., Warden, M.R., Ferenczi, E.A., Tsai, H.C., Finkelstein, J., Kim, S.Y., Adhikari, A., Thompson, K.R., Andalman, A.S., *et al.* (2013). Dopamine neurons modulate neural encoding and expression of depression-related behaviour. Nature 493, 537-541.

Vaidya, A., Mao, Z.Y., Tian, X., Spencer, B., Seluanov, A., and Gorbunova, V. (2014). Knock-In Reporter Mice Demonstrate that DNA Repair by Non-homologous End Joining Declines with Age. Plos Genetics 10.

Wu, L., Lu, Y., Jiao, Y., Liu, B., Li, S., Li, Y., Xing, F., Chen, D., Liu, X., Zhao, J., *et al.* (2016). Paternal Psychological Stress Reprograms Hepatic Gluconeogenesis in Offspring. Cell Metab 23, 735-743.

Zhang, B., Ma, S., Rachmin, I., He, M., Baral, P., Choi, S., Goncalves, W.A., Shwartz, Y., Fast, E.M., Su, Y., *et al.* (2020). Hyperactivation of sympathetic nerves drives depletion of melanocyte stem cells. Nature 577, 676-681.

Supplementary Figures

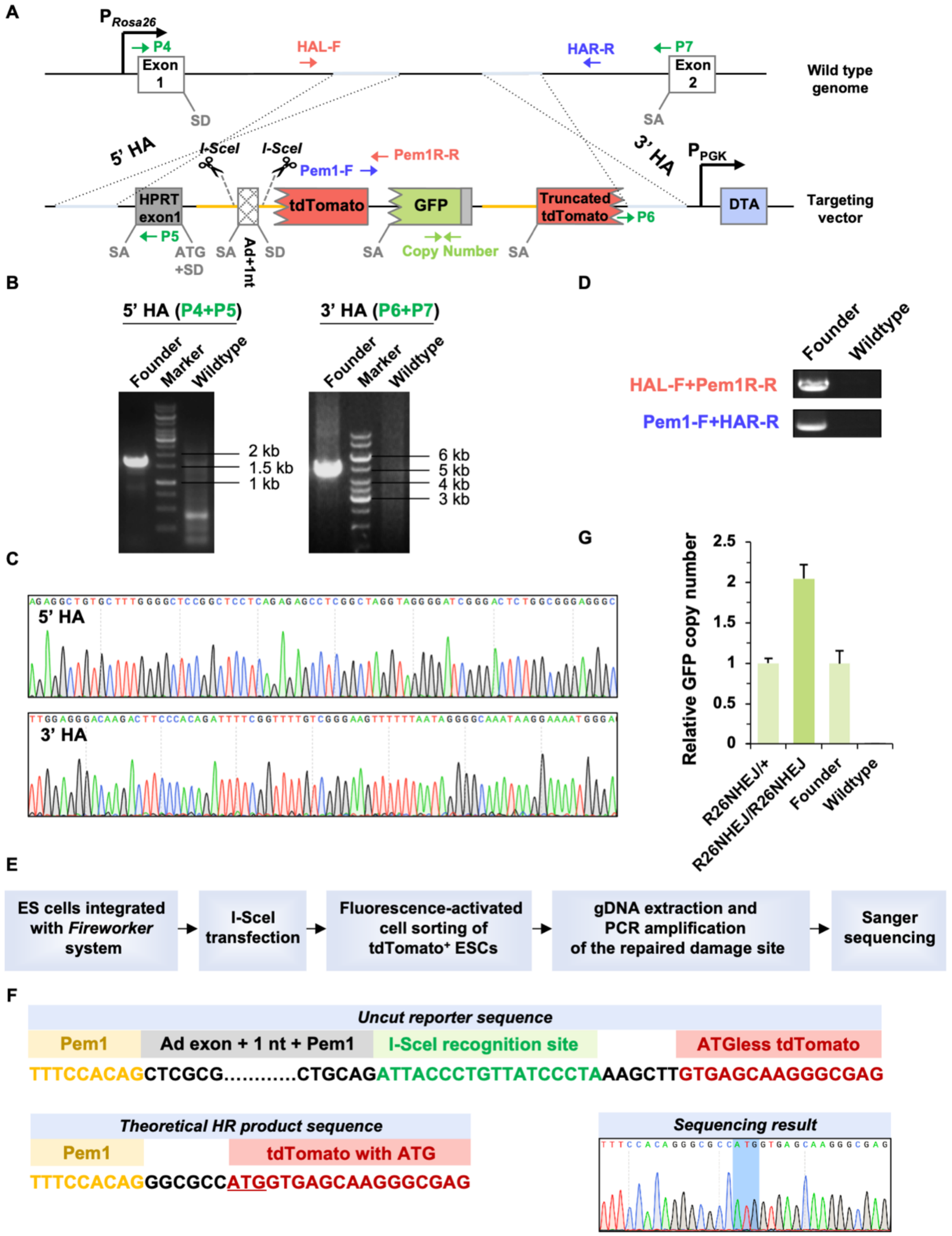

Figure S1. Characterization of the *Fireworker* mouse model.
(A) Illustration of the primers used for characterization. HA, homologous arm. **(B)** Validation of the integration at *Rosa26* locus by homologous arm PCR. **(C)** Representative Sanger sequencing result of the homologous arm PCR. **(D)** Validation of the important elements of the reporter by long-range PCR. **(E-F)** Validation of the HR repair product. The workflow was shown in (E), the sequence of uncut reporter and the HR product, and the Sanger sequencing result were shown in (F). **(G)** Determination of the copy number of the reporter in founder mice. The genomic DNA extracted from previously published homozygous and heterozygous R26NHEJ mice was used as a reference genome. Error bars represent the s.d.

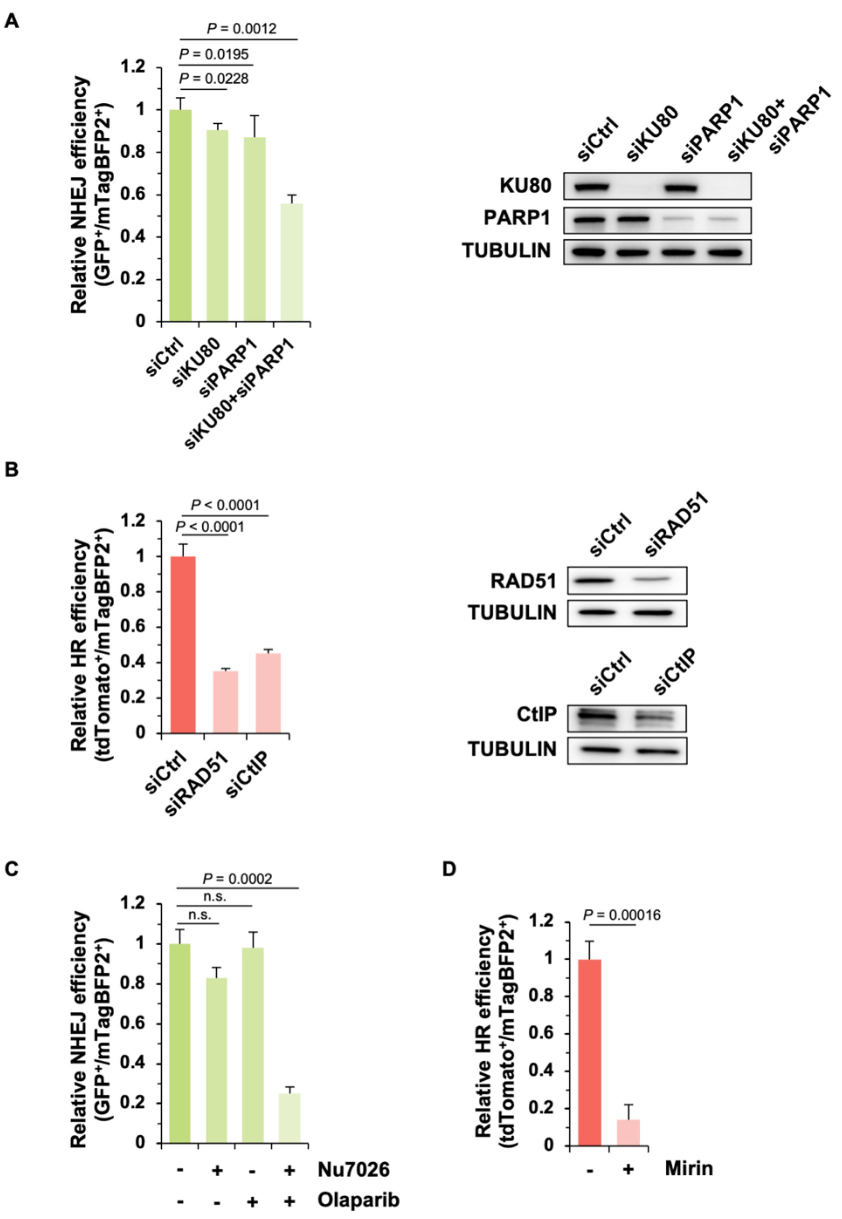

Figure S2. Validation of the *Fireworker* mouse model.

Validation of the reporter by analyzing the change in NHEJ and HR efficiency using successfully knocked-in mouse ES cells. **(A)** Effect of KU80 and PARP1 depletion on NHEJ efficiency. **(B)** Effect of RAD51 and CtIP depletion on HR efficiency. **(C)** Effect of Nu7026 (8 μM), an inhibitor of the c-NHEJ factor DNA-PKcs, and olaparib (0.5 μM), an inhibitor of the alt-NHEJ factor PARP1, on NHEJ efficiency. **(D)** Effect of 100 μM mirin, an inhibitor of HR factor Mre11, on HR efficiency. Error bars represent the s.d. Experiments were repeated at least three times. Unpaired Student's t-test was employed for significance determination.

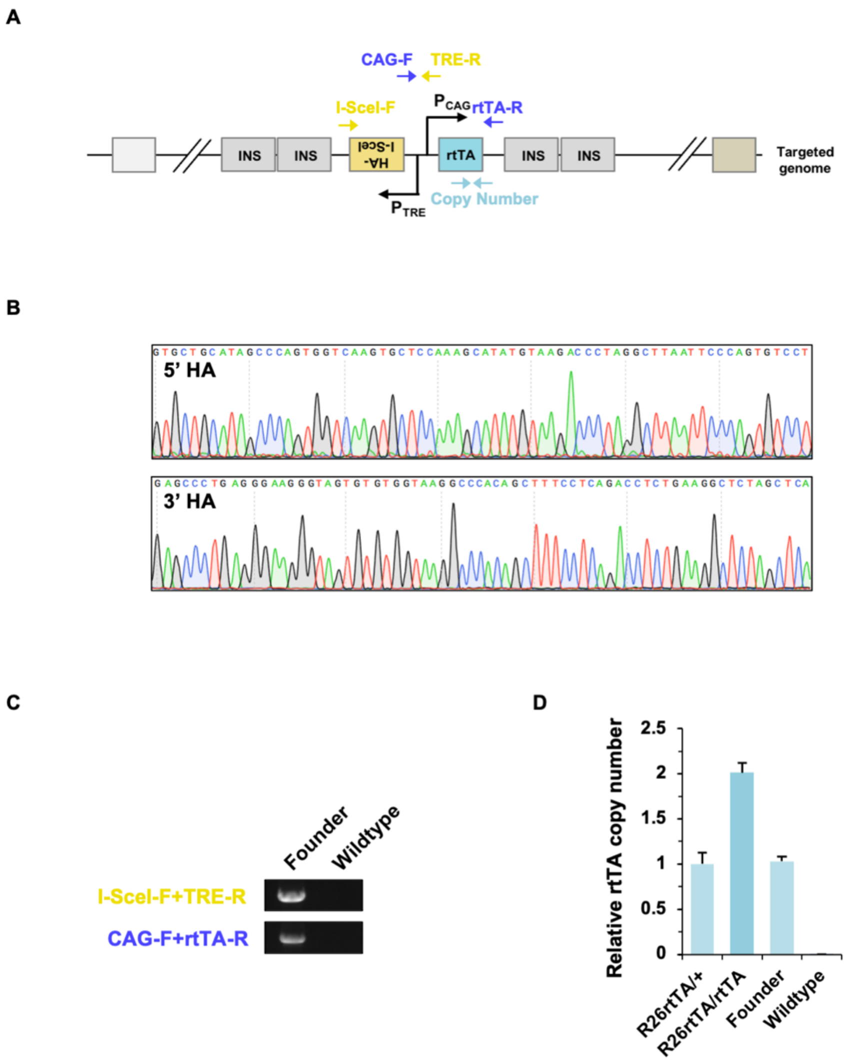

Figure S3. Characterization of the *Breaker* mouse model.
(A) Illustration of the primers used for characterization. **(B)** Representative Sanger sequencing result of the homologous arms (HA). **(C)** Validation of important elements of the reporter by long-range PCR. **(D)** Determination of the copy number of the reporter in founder mice. Genomic DNA extracted from previously published homozygous and heterozygous R26rtTA mice was used as a reference genome. Error bars represent the s.d.

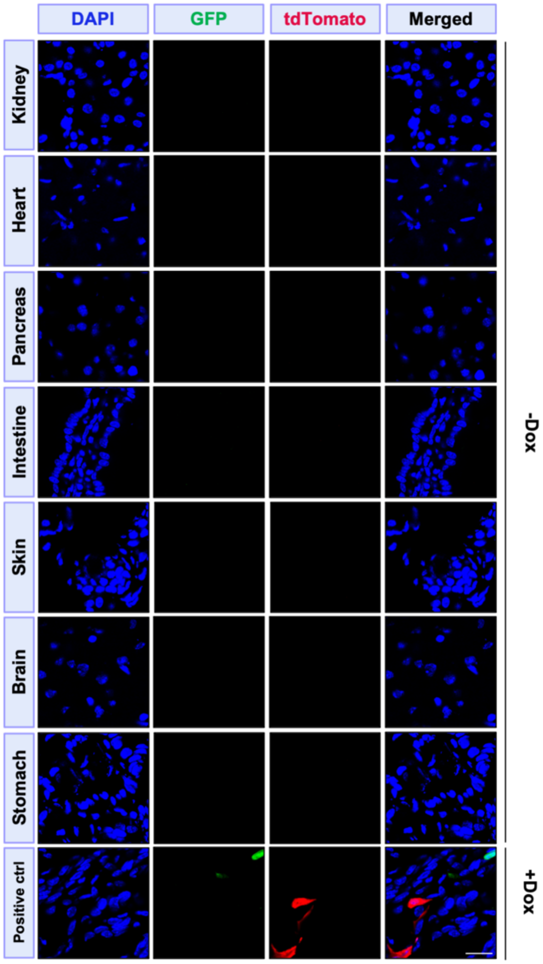

Figure S4. Analysis of GFP and tdTomato accumulation in IDDoR mice.
1-year-old IDDoR mice without doxycycline administration were sacrificed for GFP and tdTomato staining. There was no GFP and tdTomato fluorescence observed in the seven organs. The stomach from doxycycline-treated mice was used as positive control. Scale bar: 20 μm.

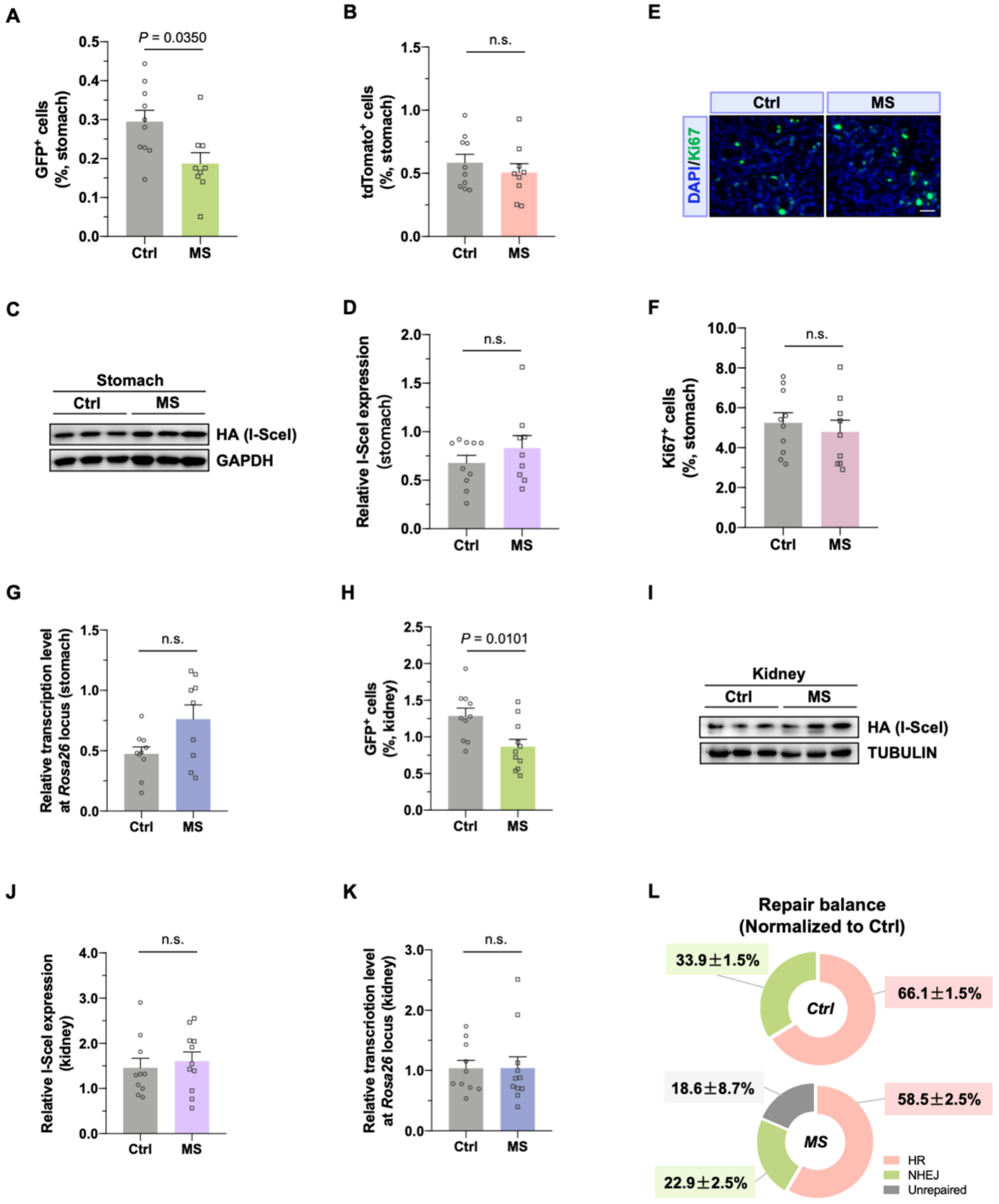

Figure S5. Mental stress-associated repair efficiency analysis using the IDDoR system.
(A-B) Percentage of GFP^+ and $tdTomato^+$ cells in stomachs; each dot represents one mouse. **(C-D)** Relative expression level of HA-tagged I-SceI in stomachs. Representative images are shown in (C), and the quantification is shown in (D). Each dot represents one mouse. **(E-F)** Ki67 staining in stomachs. Representative images are shown in (E), and the quantification result is shown in (F). Scale bar: 20 µm. **(G)** Relative transcription level of the *Rosa26* locus in stomachs. Experiments were repeated at least three times. **(H)** Percentage of GFP^+ cells in kidneys; each dot represents one mouse. **(I-J)** Relative expression level of HA-tagged I-SceI in kidneys. Representative images are shown in (I), and the quantification is shown in (J). Each dot represents one mouse. **(K)** Relative transcription level of the *Rosa26* locus in kidneys. **(L)** Donut plot of NHEJ and HR balance in stomachs. The NHEJ preference was calculated as GFP^+ cells% / (GFP^+ cells% + $tdTomato^+$ cells%), and the HR preference was calculated as $tdTomato^+$ cells% / (GFP^+ cells% + $tdTomato^+$ cells%). Data were normalized to control mice. The percentage of unrepaired break was calculated as 100% – (GFP_{MS}^+ cells% + $tdTomato_{MS}^+$ cells%) / (GFP_{Ctrl}^+ cells% + $tdTomato_{Ctrl}^+$ cells%). Experiments were repeated at least three times. Ctrl, control mice; MS, mice with mental stress. Error bars represent the s.e.m. The Mann–Whitney U test was employed for significance determination.
